# Supplementary material for: Observational descriptive study of ultrasound use and its impact on clinical decisions in the accident and emergency department at Georgetown public hospital corporation
Source: PLoS One. 2020 May 22;15(5):e0233379. doi: 10.1371/journal.pone.0233379 (PMC7244115; doi:10.1371/journal.pone.0233379)
Supplement: S1 File — (PDF) [file pone.0233379.s001.pdf]

Ultrasound usage: GPHC A&E

Physician:___________________ Date:___________ Shift: ________ Shift length: 8H or 12H

Position: Registrar Resident GMO Total patients seen on Shift: ______________

Pt	Ultrasound Type	U/S Pos or neg	If positive, findings:	Dispo change	If dispo was changed	Med change
		+ / -		Y or N	D/C - Spec Review - Admit - Theatre	Y or N
		+ / -		Y or N	D/C - Spec Review - Admit - Theatre	Y or N
		+ / -		Y or N	D/C - Spec Review - Admit - Theatre	Y or N
		+ / -		Y or N	D/C - Spec Review - Admit - Theatre	Y or N
		+ / -		Y or N	D/C - Spec Review - Admit - Theatre	Y or N
		+ / -		Y or N	D/C - Spec Review - Admit - Theatre	Y or N
		+ / -		Y or N	D/C - Spec Review - Admit - Theatre	Y or N
		+ / -		Y or N	D/C - Spec Review - Admit - Theatre	Y or N
		+ / -		Y or N	D/C - Spec Review - Admit - Theatre	Y or N
		+ / -		Y or N	D/C - Spec Review - Admit - Theatre	Y or N
		+ / -		Y or N	D/C - Spec Review - Admit - Theatre	Y or N
		+ / -		Y or N	D/C - Spec Review - Admit - Theatre	Y or N
		+ / -		Y or N	D/C - Spec Review - Admit - Theatre	Y or N
		+ / -		Y or N	D/C - Spec Review - Admit - Theatre	Y or N
		+ / -		Y or N	D/C - Spec Review - Admit - Theatre	Y or N
		+ / -		Y or N	D/C - Spec Review - Admit - Theatre	Y or N
		+ / -		Y or N	D/C - Spec Review - Admit - Theatre	Y or N
		+ / -		Y or N	D/C - Spec Review - Admit - Theatre	Y or N
		+ / -		Y or N	D/C - Spec Review - Admit - Theatre	Y or N

Ultrasound usage: GPHC A&E – Page 2

Pt	Ultrasound Type	U/S Pos or neg	If positive, findings:	Dispo change	If dispo was changed	Med change
		+ / -		Y or N	D/C - Spec Review - Admit - Theatre	Y or N
		+ / -		Y or N	D/C - Spec Review - Admit - Theatre	Y or N
		+ / -		Y or N	D/C - Spec Review - Admit - Theatre	Y or N
		+ / -		Y or N	D/C - Spec Review - Admit - Theatre	Y or N
		+ / -		Y or N	D/C - Spec Review - Admit - Theatre	Y or N
		+ / -		Y or N	D/C - Spec Review - Admit - Theatre	Y or N
		+ / -		Y or N	D/C - Spec Review - Admit - Theatre	Y or N
		+ / -		Y or N	D/C - Spec Review - Admit - Theatre	Y or N
		+ / -		Y or N	D/C - Spec Review - Admit - Theatre	Y or N
		+ / -		Y or N	D/C - Spec Review - Admit - Theatre	Y or N
		+ / -		Y or N	D/C - Spec Review - Admit - Theatre	Y or N
		+ / -		Y or N	D/C - Spec Review - Admit - Theatre	Y or N
		+ / -		Y or N	D/C - Spec Review - Admit - Theatre	Y or N
		+ / -		Y or N	D/C - Spec Review - Admit - Theatre	Y or N
		+ / -		Y or N	D/C - Spec Review - Admit - Theatre	Y or N
		+ / -		Y or N	D/C - Spec Review - Admit - Theatre	Y or N
		+ / -		Y or N	D/C - Spec Review - Admit - Theatre	Y or N
		+ / -		Y or N	D/C - Spec Review - Admit - Theatre	Y or N
		+ / -		Y or N	D/C - Spec Review - Admit - Theatre	Y or N
