# Supplementary material for: Observational descriptive study of ultrasound use and its impact on clinical decisions in the accident and emergency department at Georgetown public hospital corporation
Source: PLoS One. 2020 May 22;15(5):e0233379. doi: 10.1371/journal.pone.0233379 (PMC7244115; doi:10.1371/journal.pone.0233379)
Supplement: Attachment — Submitted filename: Ultrasound Use and its Impact_Rebuttal Letter_2.docx [file pone.0233379.s002.docx]

15 April 2020

To Whom It May Concern:

Thank you for your reviews of the submitted article, *Observational Descriptive Study of Ultrasound Use and its Impact on Clinical Decisions in the Accident and Emergency Department at Georgetown Public Hospital Corporation*. Included in this letter are responses to concerns by the initial reviewers. The original article with edits is also included with this resubmission along with the article with the changes fully included.

The requested journal requirements have been addressed as follows:

1. Adjustments have been made to the formatting. There are no directions on file naming based in the link provided.
2. In response to the data, the data has been shared in a public depository. It can be found at the following location: dx.doi.org/10.17504/protocols.io.bd6yi9fw.
3. The caption for the supplementary file is included in the supporting information section.

In regards to the Review Comments to the Author, responses are addressed as follows:

*Reviewer #1*

-Additional detail regarding the described treatment facility has been included n the manuscript.

-Specific details about the gestational ages of the pregnancies is not available to report. The issues are primarily first trimester issues and issues related to failed pregnancies (retained products of conception).

-For the EFAST exam, the primary positive findings were pneumothorax, hemothorax, and intraperitoneal bleeding. For cardiac causes, decreased left ventricular function was the predominant finding. For each application, the positive findings were incompletely recorded and therefore was not reported.

-POCUS guided procedures are very rare. Central lines kits are not always available. Thoracentesis and paracentesis are usually performed on the medicine wards where there is more physical space to perform the procedure safely.

*Reviewer #2*

-There are no questions to address from Reviewer #2.

*Reviewer #3*

-We reviewed both of the mentioned articles prior to our initial submission, Shah et al and Kotlyar and Moore. The article by Shah et al was done in rural hospitals and does not clearly state the clinical setting. Specifically, it is not in the same clinical setting as our study, the emergency department. The Kotlyar and Moore study involved all departments of the hospital. Only 28% of the patients were in the emergency department. It was not initially included for this reason, but we have added per the reviewer’s request.

- The goal of this study was to quantify the use of ultrasound in the A&E. There is an established training program for the residents. There is ongoing quality assessment of all scans performed by the GMOs and residents in real time with the registrars and visiting ultrasound-trained faculty. There are no current mechanisms for data storage and ongoing review of the registrars’ studies. Each registrar has completed the ultrasound certification.

- The availability of advanced imaging beyond ultrasound is severely limited and rarely used. The clinical decision for advanced imaging is usually made by the consulting service (ex. general surgery) because many patients cannot afford it and it takes a large amount of time.

*Reviewer #4*

-Line numbers have been inserted.

-The study design has been more clearly communicated in both the title and body of the manuscript.

-The description of the study has been updated to more accurately describe the scope of our study.

-This paper was clarified as an observational study. We have reviewed the STROBE guidelines and ensured that our manuscript fits within the recommendations. The citation has been included in the references.

-The SQUIRE guidelines are no longer applicable to this study.

-The IRB approval number has been included in the manuscript.

-The two month sample was felt to be an adequate cross section to reflect ultrasound use at GPHC. We used the similar cited studies that were performed at clinical sites in Africa to help guide our sample size.

-Statistical methods have been clarified. Simple proportions were used to report the data.

- Similar data from a U.S. training site was not available for direct comparison. In regards to non-academic sites in Guyana, there is currently no data available. GPHC is the only tertiary center. The remaining regional health facilities have very limited resource. One site just recent acquired an ultrasound device for the A&E, and training is on-going.

Thank you,

Davendra Vishaul Kissoon, MD

Jordan Rupp, MD

Corresponding Author:

Jordan Rupp, MD, FACEP

1313 21st Avenue South

Oxford House 703

Nashville, TN 37212

(t) 615-936-0087

(m) 4190377-7526

(f) 615-936-1316

jordan.rupp@vumc.org
